# Postoperative complications affect early recurrence of hepatocellular carcinoma after curative resection

**DOI:** 10.1186/s12885-015-1720-0

**Published:** 2015-10-14

**Authors:** Yan-Ming Zhou, Xiao-Feng Zhang, Bin Li, Cheng-Jun Sui, Jia-Mei Yang

**Affiliations:** 1 Department of Hepatobiliary & Pancreatovascular Surgery, First affiliated Hospital of Xiamen University, Xiamen, China; 2 Department of Special Treatment, Eastern Hepatobiliary Surgery Hospital, Second Military Medical University, Shanghai, China

**Keywords:** Hepatocellular carcinoma, Recurrence, Resection, Postoperative complications

## Abstract

**Background:**

Postoperative recurrence remains the major cause of death after curative resection for hepatocellular carcinoma (HCC). This study was conducted to evaluate the impact of postoperative complications on HCC recurrence after curative resection.

**Methods:**

The postoperative outcomes of 274 HCC patients who underwent curative resection were analysed retrospectively.

**Results:**

Of the 247 HCC patients, 103 (37.6 %) patients developed postoperative complications. The occurrence of postoperative complications was found to be associated with a significantly higher tumor recurrence (76.2 % vs. 56.6 %, *P* = 0.002) and a lower 5-year overall survival rate (27.7 % vs. 42.1 %; *P* = 0.037) as compared with those without complications. Regarding the recurrence pattern, early recurrence (≤2 years) was more frequently seen in patients with complications than that in patients without complications (54.5 % vs.38.6 %; *P* = 0.011). Multivariate analysis indicated that postoperative complications occurrence was an independent risk factor for early recurrence (odds ratio [OR] 2.223; 95 % confidence intervals [95 % CI] 1.161–4.258, *P* = 0.016) and poor overall survival (OR 1.413; 95 % CI, 1.012–1.971, *P* = 0.042).

**Conclusions:**

The results of the present study indicate that the occurrence of postoperative complications is a predictive factor for HCC recurrence after curative hepatectomy, especially for early recurrence.

## Background

Hepatocellular carcinoma (HCC) is the 5th most common malignancy and ranks the 3rd cause of cancer-related death worldwide. Although hepatic resection is an effective treatment option for HCC, the long-term prognosis remains poor in most series mainly because of the high tumor recurrence in the remnant liver [1].

With improvements in careful patient selection, surgical techniques and perioperative care, hepatectomy for HCC has become a safe procedure with a reported operative mortality rate lower than 5 % at high-volume centers. However, the incidence of complications is as high as 30.9–42.6 % [2–4]. Several studies have assessed the impact of postoperative complications on long-term survival in patients with HCC [2, 5–7], but few data are available in the literature regarding its impact on the risk of tumor recurrence. To clarify this issue, we conducted a retrospective study of 274 consecutive patients who underwent curative resection for HCC.

## Methods

### Patients

Included in this study were 274 consecutive patients who underwent curative resection for HCC at the Department of Special Treatment of the Eastern Hepatobiliary Surgery Hospital affiliated to the Second Military Medical University (Shanghai, China) between January 2004 and September 2008. Curative resection was defined as complete macroscopic removal of the tumor with a microscopic free margin. Patient selection for hepatectomy and details of hepatectomy were as previously reported [8]. A major resection was defined as removal of at least three liver segments, and a minor resection was defined as removal of two or fewer than two segments. Postoperative mortality was defined as any death occurring within 30 days of surgery or within the same hospital stay. Postoperative complications were defined as occurrence of any medical or surgical complication during the hospital stay. Data for long-term outcomes, including overall survival (OS) and recurrence, were obtained by reviewing the medical records at the last follow-up. After surgery, patients were followed-up every 1 month by tumor marker (alpha-fetoprotein, AFP) analysis and ultrasound at least every 3 months in the first year after hepatectomy, and then at gradually increasing intervals. When tumor recurrence or metastases were suspected, further investigations with computed tomography scan, or magnetic resonance imaging were done. Fine needle aspiration/biopsies were done when necessary. Recurrences were divided as early (≤2 years) and late (>2 years) recurrences [9]. This study was approved by the ethics committee of the Second Military Medical University, and all participants provided written informed consent.

### Statistical analysis

Categorical and continuous data were compared by the *χ*2 test and the Student *t* test, respectively. Overall survival was determined by Kaplan-Meier analysis. The Cox proportional hazard regression model was used to determine the independent risk factors for recurrence and survival as well as complication, based on the variables selected by univariate analysis. All statistical analyses were performed using SPSS for Windows (version 11.0; SPSS Institute, Chicago, IL, USA). *P* < 0.05 was considered statistically significant.

## Results

### Patient characteristics

The 274 HCC patients included 253 (92.3 %) men and 21 (7.7 %) woman with a median age of 56 (range 21–87) years. Of them, 103 (37.6 %) developed 169 postoperative complications (Table 1), of which pleural effusion (18.9 %) and ascites (12 %) were the most common complications. One patient died of postoperative sepsis and another one died of postoperative liver failure, giving an overall hospital mortality rate of 0.8 %.

**Table 1:** Details of postoperative complications

Types of complications	No. of patients (%)
Liver failure/insufficiency	14 (5.1)
Bile leak	5 (1.8)
Biloma/abscess	2 (0.7)
Intra-abdominal infection	5 (1.8)
Hemorrhage	4 (1.5)
Ascites	31 (12)
Wound infection/dehiscence	11 (4)
Pleural effusion	52 (18.9)
Pneumonia	11 (4)
Atelectasis	8 (2.9)
Arrhythmia	7 (2.5)
Heart failure	1 (0..3)
Urinary retention	4 (1.5)
Urinary tract infection	3 (1.1)
Renal insufficiency/failure	3 (1.1)
Ileus	5 (1.8)
Delayed Gastric Emptying	3 (1.1)

The clinicopathological characteristics of the patients with and without postoperative complications are shown in Table 2. The percentages of elderly patients and those with larger tumors were significantly higher in the postoperative complication group than those in the non-complication group. Major resections were performed more frequently in patients with postoperative complications. Similarly, those with postoperative complications had a longer operation time, more intraoperative blood loss and a greater blood transfusion requirement.

**Table 2:** Comparison of clinicopathological features between patients with or without postoperative complication

Variable	With complications (*n =* 103)	Without complications (*n =* 171)	*P*-value
Sex (male/female)	95/8	158/13	0.960
Age (years)	58.3 ± 11.2	54.7 ± 10.2	< 0.001
Hepatitis B infection	97 (94.2 %)	166 (97.1 %)	0.236
Hepatitis C infection	0	1 (0.6)	0.437
Child-Pugh (A/B)	98/5	168/3	0.140
AFP level > 400 ng/ml	27 (26.2 %)	31 (18.1 %)	0.113
Cirrhosis	41 (39.8 %)	76 (44.4 %)	0.452
Tumor diameter > 5 cm	73 (68.9 %)	77 (51.5 %)	0.005
Tumor number (St/Mt)	85/18	147/24	0.444
Tumor capsule absent	49 (47.6 %)	72 (42.1 %)	0.377
Vascular invasion	53 (51.5 %)	72 (42.1 %)	0.132
Edmondson’s grade (I-II/III-IV)	22/ 81	34/137	0.769
TNM stage (I-II/III)	61/42	115/56	0.179
Operative procedure (MAR/MIR)	59/44	76/95	0.040
Use of the Pringle’s maneuver	97 (94.2 %)	160 (93.6 %)	0.840
Operation time (min)	297 ± 113	256 ± 134	< 0.001
Intraoperative blood loss (ml)	840 ± 520	620 ± 470	< 0.001
Blood transfusion requirement	34 (33 %)	37 (21.6 %)	0.037

*St* single tumor, *Mt* multiple tumors, *AFP* alpha-fetoprotein, *MAR* major resection, *MIR* minor resection

### Patient recurrence and survival

During a median follow-up period of 38 months, 175 (64.3 %) patients (excluding the hospital deaths) experienced intrahepatic recurrences by the end of the study period. The initial management for the recurrences included transarterial chemoembolization (*n =* 72), percutaneous ablation (*n =* 37), repeated resection (*n =* 28), liver transplantation (*n =* 3), and conservative treatment (*n =* 35). The frequency of HCC recurrence was 77 (76.2 %) in patients with postoperative complications and 98 (56.6 %) in those without postoperative complications (*P* = 0.002). There were 121 early and 54 late recurrences. Early recurrence was more frequently seen in patients with postoperative complications than that in patients without postoperative complications (54.5 % vs.38.6 %; *P* = 0.011), while late recurrence was similar between the two groups (22.8 % vs.18.1 %; *P* = 0.354).

Univariate analysis showed that significant risk factors associated with early recurrence were serum AFP > 400 ng/mL, tumor diameter > 5 cm, the absence of tumor capsules, vascular invasion, multiple tumors, an advanced TNM stage, and occurrence of postoperative complications. Multivariate analysis showed that postoperative complications were one of the independent factors (odds ratio [OR] 2.223; 95 % confidence intervals [95 % CI] 1.161-4.258, *P* = 0.016) (Table 3).

**Table 3:** Multivariable analysis of risk factors for early recurrence

Variables	Odds ratio	95 % confidence intervals	*P*-value
Alpha-fetoprotein > 400 ng/mL	1.613	1.101–2.362	0.014
Tumor diameter > 5 cm	1.083	0.736–1.593	0.685
Tumor capsule absent	0.747	0.452–1.235	0.255
Vascular invasion	2.916	1.541–5.516	< 0.001
TNM stage (III)	1.353	0.962–1.902	0.082
Multiple tumors	1.204	1.011–1.433	0.037
Postoperative complications	2.223	1.161–4.258	0.016

**Fig. 1:**
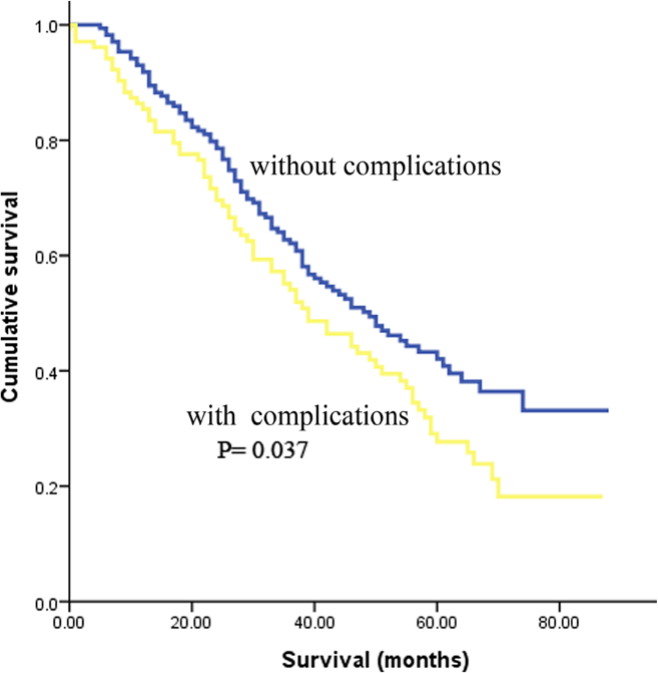
Cumulative overall survival after surgical resection in patients with and without postoperative complications

The 5-year OS for the entire cohort of HCC patients was 37 %, and the 5-year OS in patients who experienced postoperative complications was significantly lower than that in patients without complications (27.7 % vs. 42.1 %; *P* = 0.037) (Fig. 1). Multivariate analysis demonstrated that cirrhosis (OR 1.544; 95 % CI 1.051–2.269, P = 0.027), vascular invasion (OR 2.712; 95 % CI, 1.371–5.364, *P* = 0.004), and postoperative complications (OR 1.413; 95 % CI, 1.012–1.971, *P* = 0.042) were significant independent risk factors for decreased OS.

### Risk factor for postoperative complications

Univariate analysis showed that old age (≥70 years), major resections, large tumor, blood transfusion, operating time ≥ 300 min, and intraoperative blood loss ≥1000 ml were associated with the occurrence of postoperative complications. Multivariate analysis identified two independent risks for complications occurrence: old age (OR 2.173; 95 % CI, 1.014–4.656, *P* = 0.046) and intraoperative blood loss ≥1000 ml (OR 1.771; 95 % CI, 1.138–2.756, *P* = 0.011).

## Discussion

Complications after hepatectomy for HCC are common with morbidity rates between 30.9 and 42.6 % [2–4]. In line with literature, a morbidity rate of 37.6 % was observed in the present study. The most common were ascites or pleural effusion. The high incidence of ascites was probably related to high portal pressure after liver resection, which can trigger massive ascites by stimulating neurohormonal systems to promote renal water and sodium resorption [10]. The changes generated by vascular clamping and instruments working close to the diaphragm during hepatectomy may increase the risk of pleural effusion [11].

There is no doubt that postoperative complications may lead to prolonged hospital stay and higher medical costs. Recent evidence has suggested that postoperative complications also have negative impact on long-term survival in patients with gastric cancer [12], esophageal cancer [13], pancreatic cancer [14], hilar cholangiocarcinoma [15], and colorectal liver metastasis [16, 17]. To the best of our knowledge, there have been four studies on the effect of postoperative complications on the survival and prognosis of HCC patients. In a study of 863 HCC patients undergoing curative resection, Chok et al. [5] found that the presence of postoperative complications was independently associated with poor OS. In another study of 291 patients, Kusano et al. [2] reported that the 5-year OS rate was significantly lower in patients with perioperative complications than that in those without these complications (34.3 % vs.48.7 %). Their multivariate analysis showed that the presence of perioperative complications was an independent predictor of poor OS. A study of 100 patients by Mizuguchi et al. [6], and a study of 376 patients by Okamura et al. [7] also reported the similar results. Consistent with previous investigations, our study reconfirmed the prognostic value of postoperative complications.

Few data are available in the literature regarding the potential impact of complications on HCC recurrence. It was found in this study that postoperative complications were associated with a higher HCC recurrence after hepatectomy, and identified as an independent risk factor for early recurrence. This finding is consistent with the finding of Okamura et al. [7], who reported that the recurrence-free survival curve for patients with postoperative complications was steeper than that for the group without, especially from 12 to 24 months. Early recurrence might reflect residual micrometastasis in the liver [9]. In the field of periampullary cancer surgery, the negative impact of postoperative complications on survival is even more prominent in patients with microscopically residual disease [18]. An explanation for negative impact of postoperative complications on cancer recurrence remains unclear. One possible factor promoting metastatic growth and early recurrence is immunosuppression resulting from systemic inflammatory responses [16]. It has been demonstrated that surgical trauma and septic inflammation may promote T-cell commitment toward a T-helper 2-type lymphocyte pattern [19]. The T-helper 2-type cytokines (interleukin-10 in particular) were shown to down-modulate tumor-specific immune response probably through several mechanisms: (i) directly suppressing interferon γ and interleukin-12 production, thereby preventing the activation of cytotoxic T lymphocytes and natural killer cells; (ii) reducing major histocompatibility complex expression on the surface of tumor cells, thus preventing the optimal expression of binary complexes formed by tumor antigen in association with major histocompatibility complex molecules on the surface of such cells; and (iii) inhibiting tumor antigen presentation by antigen-presenting cells [20]. In fact, serum interleukin-10 level was found to be associated with a worse disease-free survival in patients with resectable HCC [21]. As late recurrence is considered as the most significant factor attributable to hepatitis related multicentric carcinogenesis, it is therefore reasonable to conclude that postoperative complications have no effect on late recurrence.

Conventional aggressive biological behaviors of HCC such as vascular invasion, poor tumor differentiation and multiple tumors have been reported to affect recurrence. Our finding presented additional data that the surgeon’s performance is also an important determinant. Similar to other series [2], our study also found that increased intraoperative blood loss was an independent predictor of complications occurrence after hepatic resection for HCC. Various methods of vascular occlusion (such as Pringle’s maneuver, total or selective hepatic vascular exclusion, combination of Pringle’s maneuver and infrahepatic inferior vena cava clamping, and hanging maneuver) and new surgical devices (such as Harmonic Scalpel, radiofrequency electrodes, and staplers) have been suggested for reducing blood loss during liver parenchymal transaction. Some recent studies [22] have suggested that laparoscopic surgery for HCC can reduce intraoperative blood loss without compromising the oncologic outcome. Hepatic surgeons should know how to use these surgical strategies appropriately according to the actual situation in clinical practice.

Unlike previous findings [7], this study failed to show that the use of Pringle’s maneuver is a risk factor for postoperative complications. The main drawback related to Pringle’s maneuver is the hepatic ischemia-reperfusion injury. However, there is ample evidence that liver parenchyma tolerates prolonged ischemic injury better than the effect of massive bleeding and blood transfusion [23]. More importantly, there is no clinical evidence that Pringle’s maneuver adversely affects the oncologic outcome [24]. To avoid irreversible injury to the liver, Pringle’s maneuver should be done with intermittent clamping with an upper limit of 120 min [23].

It was also found in our study that old age was a significant risk factor for postoperative complications occurrence, probably because elderly patients are more likely to have co-morbidities. This emphasizes the need of multidisciplinary preoperative evaluation and postoperative management of elderly patients undergoing hepatic resection [25].

There are several limitations in this study. First, its retrospective nature may inherently incur selection bias. In addition, as this is a single-center study, the generalizability of our findings awaits further investigation. Finally, the effect of classification of postoperative morbidity by the Clavien-Dindo stratification was not analyzed because of the limited number of patients [26]. Farid et al. [16] reported a negative relationship between postoperative morbidity and the oncologic outcome in 750 patients undergoing hepatic resection for colorectal metastases. Interestingly, the severity of complications was found to be irrelevant to the oncologic outcome. A similar observation was also noted by Correa-Gallego et al. [17]. Therefore, any postoperative complications (even minor ones) need to be prevented for the sake of improving the cancer-specific outcome [17].

## Conclusion

The results of the present study indicate that the occurrence of postoperative complications is a predictive factor for HCC recurrence after curative hepatectomy, especially for early recurrence. To improve the patients’ oncological outcome, surgeons should do their best to minimize the occurrence of postoperative complications wherever possible. Further large prospective multicenter clinical trials are necessary to confirm our results.

## Abbreviations

HCC: Hepatocellular carcinoma
OR: Odds ratio
95 % CI: 95 % confidence intervals
OS: overall survival
TNM: Tumor node metastasis
